# Effect of a structurally modified human granulocyte colony stimulating factor, G-CSFa, on leukopenia in mice and monkeys

**DOI:** 10.1186/1756-8722-4-28

**Published:** 2011-06-13

**Authors:** Yongping Jiang, Wenhong Jiang, Yuchang Qiu, Wei Dai

**Affiliations:** 1 Fanzhou Biopharmagen Corporation, Suzhou, China; 2 New York University School of Medicine, Tuxedo, NY, USA

## Abstract

**Background:**

Granulocyte colony stimulating factor (G-CSF) regulates survival, proliferation, and differentiation of neutrophilic granulocyte precursors, Recombinant G-CSF has been used for the treatment of congenital and therapy-induced neutropenia and stem cell mobilization. Due to its intrinsic instability, recombinant G-CSF needs to be excessively and/or frequently administered to patients in order to maintain a plasma concentration high enough to achieve therapeutic effects. Therefore, there is a need for the development of G-CSF derivatives that are more stable and active in vivo.

**Methods:**

Using site-direct mutagenesis and recombinant DNA technology, a structurally modified derivative of human G-CSF termed G-CSFa was obtained. G-CSFa contains alanine 17 (instead of cysteine 17 as in wild-type G-CSF) as well as four additional amino acids including methionine, arginine, glycine, and serine at the amino-terminus. Purified recombinant G-CSFa was tested for its in vitro activity using cell-based assays and in vivo activity using both murine and primate animal models.

**Results:**

In vitro studies demonstrated that G-CSFa, expressed in and purified from *E. coli*, induced a much higher proliferation rate than that of wild-type G-CSF at the same concentrations. In vivo studies showed that G-CSFa significantly increased the number of peripheral blood leukocytes in cesium-137 irradiated mice or monkeys with neutropenia after administration of clyclophosphamide. In addition, G-CSFa increased neutrophil counts to a higher level in monkeys with a concomitant slower declining rate than that of G-CSF, indicating a longer half-life of G-CSFa. Bone marrow smear analysis also confirmed that G-CSFa was more potent than G-CSF in the induction of granulopoiesis in bone marrows of myelo-suppressed monkeys.

**Conclusion:**

G-CSFa, a structurally modified form of G-CSF, is more potent in stimulating proliferation and differentiation of myeloid cells of the granulocytic lineage than the wild-type counterpart both in vitro and in vivo. G-CSFa can be explored for the development of a new generation of recombinant therapeutic drug for leukopenia.

## Background

Granulocyte colony stimulating factor (G-CSF) is the principal cytokine that regulates survival, proliferation, and differentiation of neutrophilic granulocyte precursors [1-3], and it functionally activates mature blood neutrophils as well [4-7]. Among the family of colony-stimulating factors, G-CSF is the predominant inducer of terminal differentiation of granulocytes [8]. Recombinant human G-CSF has been used as a therapeutic drug for leukopenia of cancer patients who receive myelo-suppressive radio-or chemotherapy [9,10]. In recent years, recombinant G-CSF has also been used for the treatment of congenital neutropenia and stem cell mobilization [11,12]. It has been more than fifteen years since recombinant G-CSF was successfully used in the clinics. Due to its intrinsic instability, G-CSF needs to be excessively and/or frequently administered to patients in order to maintain a plasma concentration high enough to achieve therapeutic effects. This administration regimen not only causes inconvenience and pains in patients but also increases the chance for infections. Therefore, there is a necessity for the development of G-CSF derivatives that are more stable and active in vivo. Here, we report that G-CSFa, a recombinant G-CSF derivative, exhibits potent biological activities both in vivo and in vitro and that these activities appear to result from an enhanced stability of modified G-CSF and its binding affinity to the cognate receptor.

## Methods

### Animals

Male BALB/CICR C57 mice with an average weight of 22.5 ± 1.2 g (20.0 ~ 24.9 g), and monkeys with an average weight of (5.4 ± 1.0 kg) were selected for our studies. Animals were housed in individual stainless steel cages in a study room with a regulated temperature of 24 ± 2°C, relative humidity of 50 ± 10%, and a 12-h light cycle. All animal experiments were conducted in compliance with the Guidelines for Animal Experimentation issued by the Chinese Association for Laboratory Animal Science and the Standards Relating to the Care and Management of Experimental Animals throughout the study.

### Mutant G-CSF and Expression of G-CSFa in E. Coli

G-CSF cDNA was obtained through reverse transcriptase-mediated polymerase chain reaction (RT-PCR) using total RNAs isolated from human monocytes. The primers used for PCR were as follows: upstream primer, 5' TGG ATC CAT GAC CCC CCT GGG CCC 3' and downstream primer, 5' TAA GAT CTC AAG CTT TCA GGG CTG CGC AAG GTG GCG TA3'. The amplified products were fractionated on agarose gels. The G-CSF cDNA eluted from the agarose gel was digested by Bam HI and Hind III, and ligated to plasmid pQE3 that had been cut with the same restriction enzymes. The ligation mixture was transformed into *Escherichia coli *JM109 competent cells for characterization of the cloned cDNA. Mutant G-CSF (G-CSF^C17A^) was made by replacing codon TGC with codon GCC through site-direct mutagenesis. The identity of G-CSF cDNA, as well as the introduced mutation, was confirmed by a thorough DNA sequencing analysis. The pQE3 plasmid expressing mutant G-CSF (G-CSFa) was transformed into *E. coli *M15 cells for expression. Expression of G-CSFa was induced by isopropylthio-β-d-galactoside (IPTG).

### Refolding and purification of G-CSFa

*E. Coli *pellets were disrupted with addition of lysozyme (5 mg/liter culture) in 0.1 M TrisHCl buffer (pH 8.0). Inclusion body was collected by washing three times with an extraction buffer [50 mM TrisHCl (pH 8.0), 2 mM EDTA, 2 M urea] and it was dissolved in a buffer containing a high concentration of urea [50 mM TrisHCl (pH 8.0), 2 mM EDTA, 8 M urea, 2% DTT]. Refolding of recombinant G-CSFa was achieved by dialysis against 50 mM TrisHCl buffer (pH 8.0) for three times (12 h intervals). Refolded recombinant G-CSFa was purified by anion exchange chromatography and size exclusion chromatography. Recombinant G-CSFa was stored in 50 mM acetic acid-sodium acetate buffer (pH 5.4) containing 5% mannitol. Purified protein was also subjected to protein sequencing analysis using the Edman degradation method [13].

### Western blotting

Protein samples fractionated on denaturing (SDS) polyacrylamide gels (4% stacking gel, 12% separating gel) were transferred to a polyvinylidene difluoride (PVDF) membrane. The membrane was blocked in a 2% bovine serum albumin (BSA) solution for 1 hr and then incubated for 1 hr with a monoclonal antibody to G-CSF (R & D systems). After washing three times with a TrisHCl buffer, the membrane was incubated for 1 hr with a goat-anti-mouse immunoglobulin G (IgG) conjugated with alkaline phosphatase. Specific signals on the membrane were visualized by addition of substrate, O-phenylene diamine (OPD).

### In vitro bioactivity assay

In vitro activity of recombinant G-CSFa was determined using the murine myelobalstic cell line NFS-60 as originally described by Shirafuji [14]. We also employed this bioassay method as described above for measuring the activity of human G-CSF using NFS-60 cells. Recombinant G-CSF made in house as well as commercial one were used as positive controls.

### Animal neutropenia models

BALB/CICR C57 male mice with an average weight of 22.5 ± 1.2 g were irradiated with cesium-137 (4 Gy) using Gammacell-40 apparatus (Nurolion, Canada) to induce leukopenia. To induce leukopenia in Monkeys, animals were intravenously administered with cyclophosphamide at a dose of 50 mg/kg/day for 2 days.

### Measurement of mice bone marrow DNA content

Mouse femur was cleaned and washed with 5 mM CaCl_2_. Bone marrow cells were flushed out with a 10 ml 5 mM CaCl_2 _solution. Bone marrow cells were placed at 4°C for 30 min and then centrifuged (2,500 RPM × 15 min). The pellet was resuspended in 5 ml 0.2 M HClO, heated at 90°C for 15 min, and filtrated through a 0.45 μm filter after cooling. DNA content was determined by measuring the absorbance of the solution at 260 nm (A260_nm_) in a spectrophotometer.

### Cytology

Monkey bone marrow was aspirated from the posterior iliac crest. Bone marrow slides were prepared in a fashion similar to the blood smears, which were subjected to routine Wright's staining. Peripheral white blood cells and neutrophils were counted using an automated hematology cell counter (Biochem Immunosystem).

### Statistical analysis

Data obtained from mouse studies were subjected to statistical analysis using a Q test. Data obtained from monkey studies were subjected to statistical analysis using a Newman-Keuls test. The results were considered statistically significant when P value was less than 0.05.

## Results

**Figure 1:**
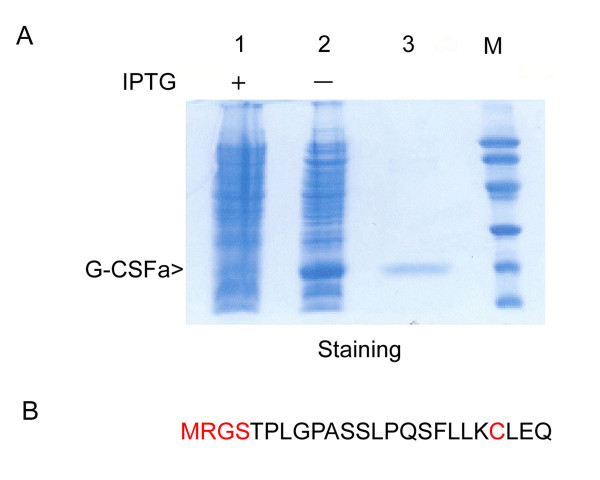
**Analysis of expression and purification of recombinant G-CSFa**. (A) G-CSFa was expressed and purified as described in Materials and Methods. Purified G-CSFa (lane 3) and bacterial cell lysates before (lane 1) and after (lane 2) IPTG addition were analyzed by SDS-PAGE. Lane M stands for molecular markers. Each experiment was repeated for at least three times and representative data are shown. (B) N-terminal amino acid sequences of G-CSFa determined by Edman degradation method. The mutated amino acid residues are highlighted in red.

**Figure 2:**
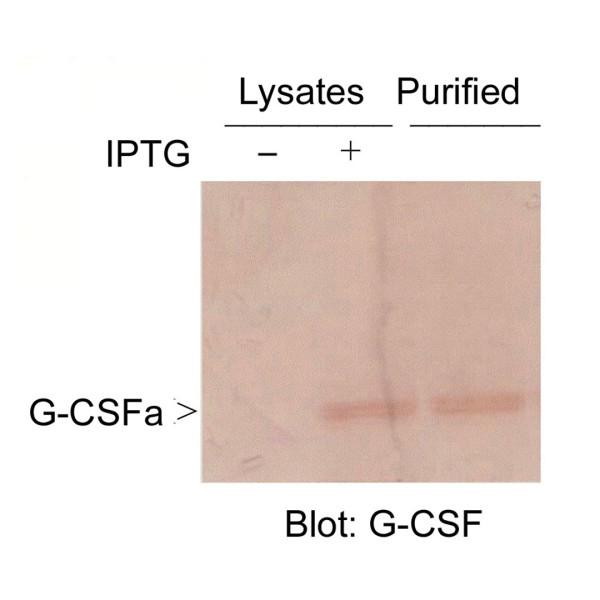
**Immunoblot analysis of purified recombinant G-CSFa**. Purified G-CSFa was blotted with the antibody to G-CSF (lane 3). Lane 1, negative control (bacterial cell lysates without IPTG induction). Lane 2, bacterial cell lysates with IPTG induction. Each experiment was repeated for at least three times and representative data are shown.

Structurally modified G-CSF (G-CSFa) was expressed in *E. coli *using a pQE vector expression system. Following the addition of IPTG, recombinant G-CSFa was highly induced (Figure 1). In fact, G-CSFa was the most predominant protein in the bacterial cell lysates after induction. Recombinant G-CSFa was subjected to extensive purification using a combination of biochemical approaches. SDS-PAGE analysis revealed that recombinant G-CSFa was purified to homogeneity and remained intact (Figure 1A). Immunoblotting analysis showed that the G-CSF antibody detected IPTG-induced G-CSFa in the total bacterial lysates as well as its the purified form (Figure 2), suggesting that the amino acid addition and substitution do not significantly change the overall conformation of protein. Protein sequencing analysis confirmed that the purified protein was the modified form of G-CSF with the addition of methionine, arginine, glycine, and serine residues at the amino-terminus and with cysteine-17 replaced by alanine as predicted (Figure 1B).

**Figure 3:**
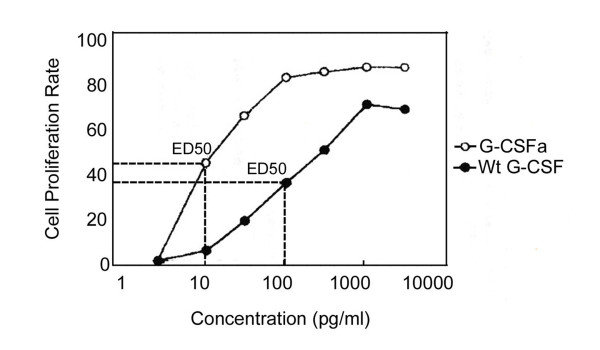
**A comparison of in vitro activity between G-CSFa and wild-type G-CSF**. Recombinant G-CSFa and G-CSF at indicated concentrations (10 pg, 20 pg, 100 pg, 200 pg, 1 μg, and 20 μg per milliliter, respectively) were used for the stimulation of NFS-60 cell proliferation. The concentrations that stimulate 50% cell proliferation rate (ED50) were obtained for each cytokine. Each experiment was repeated for at least three times and similar results were obtained.

To determine the in vitro activity of purified G-CSFa, we employed a cell proliferation assay using NFS-60 cells as described [14]. We observed that the addition of G-CSFa greatly stimulated the proliferation rate of NFS-60 cells (Figure 3). G-CSFa was more potent in stimulating the proliferation of NFS-60 cells than the wild-type recombinant G-CSF at the same concentrations (Figure 3). In fact, ED50 for G-CSFa was about 10-fold lower than that for wild-type G-CSF.

**Figure 4:**
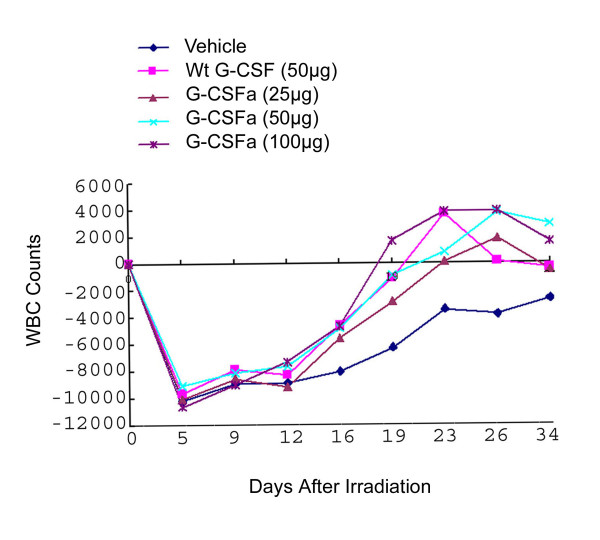
**Neutrophil recovery in irradiated mice administered with G-CSFa and wild-type G-CSF**. Groups of mice (n = 12) irradiated with cesium-137 for five days were administered daily with G-CSFa or G-CSF at the indicated doses. Mean white blood cell (WBC) counts were obtained at day 5, day 9, day 12, day 16, day 19, day 23, day 26, and day 34 after irradiation. The data were summarized from two independent experiments.

We next determined the in vivo activity of G-CSFa using the murine model. Irradiated mice were injected with wild-type G-CSF or with G-CSFa for 5 days as described in Materials and Methods. Peripheral white blood cell counts were determined at various times after cytokine injection. We observed that peripheral leukecytes in mice decreased drastically after irradiation and gradually increased to about 2/3 of the original level during the course of three weeks. Similar to that of G-CSF, G-CSFa was effective in stimulating the recovery of white blood cells in the irradiated mice (Figure 4; Table 1). At 50 μg/ml concentration, G-CSFa, but not G-CSF, was able to sustain an elevated white blood cell counts at day 26 and beyond. At day 34, which was six days after the cessation of cytokine administration, the white blood cell counts in peripheral blood of mice injected with G-CSFa remained at 128% (100 μg/ml) and 113% (50 μg/ml) of the level before irradiation, respectively. On the other hand, wild-type G-CSF was unable to support the full recovery of white blood cells to the pre-irradiation level by day 26 and beyond (Figure 4; Table 1). Bone marrow cellularity was determined by measuring the total DNA content. After irradiation for 8 days, the total DNA content of the marrow cells from mice injected with G-CSFa or G-CSF was significantly higher than that injected with vehicle although there was no significant difference in the DNA content between mice injected with G-CSF or G-CSFa (Table 2).

**Table 1:** Effect of G-CSFa on white blood cell counts in irradiated C57 Mice (10^9^/L; x ± SD; n = 12)

Groups	Before Treatment	Days After Irradiation							
		
		d5	d9	d12	d16	d19	d23	d26	d34
**Control**	11413 ± 2089	1233 ± 553	2450 ± 1104	2533 ± 850	3342 ± 811	5083 ± 260	7925 ± 1667	7575 ± 1858	8750 ± 2203

**Wt G-CSF (50 mg/kg)**	10996 ± 2731	1342 ± 337	3100 ± 1408	2667 ± 502	6358 ± 1398	9858 ± 2333^a^	14650 ± 2861^b^	11092 ± 2319^a^	10592 ± 1547

**G-CSFa (25 mg/kg)**	11083 ± 2724	855 ± 342	2508 ± 923	1900 ± 972	5483 ± 973	8192 ± 2721	11192 ± 4593^a^	12917 ± 2921^b^	10533 ± 1741

**G-CSFa (50 mg/kg)**	10178 ± 4014	1100 ± 412	2042 ± 687	2508 ± 565	5325 ± 1139	9250 ± 3766^a^	10975 ± 3052^a^	13950 ± 3087^b^	13075 ± 3120^a^

**G-CSFa (100 mg/kg)**	11814 ± 3802	1175 ± 344	2783 ± 1233	4483 ± 1394	6325 ± 1726	13467 ± 4719^b^	15650 ± 3572^b^	15700 ± 4278^b^	13383 ± 2696

a: *p *< 0.05, b: *p *< 0.01 vs. Control Group

**Table 2:** DNA content of mouse bone marrow cells

Groups	OD_260 nm_
**Vehicle**	0.65 + 0.12

**Wt G-CSF (50 μg/kg)**	1.01 + 0.34*

**G-CSFa (25 μg/kg)**	0.95 + 0.13*

**G-CSFa (50 μg/kg)**	1.01 + 0.14*

**G-CSFa (100 μg/kg)**	1.02 + 0.20*

Irradiated (4 Gy) mice were administered daily via s.c. with cytokine or vehicle as indicated. Left femur was obtained for bone marrow cell collection. These cells were subsequently processed for analysis of total DNA contents. *: *p *< 0.01 vs. vehicle

**Figure 5:**
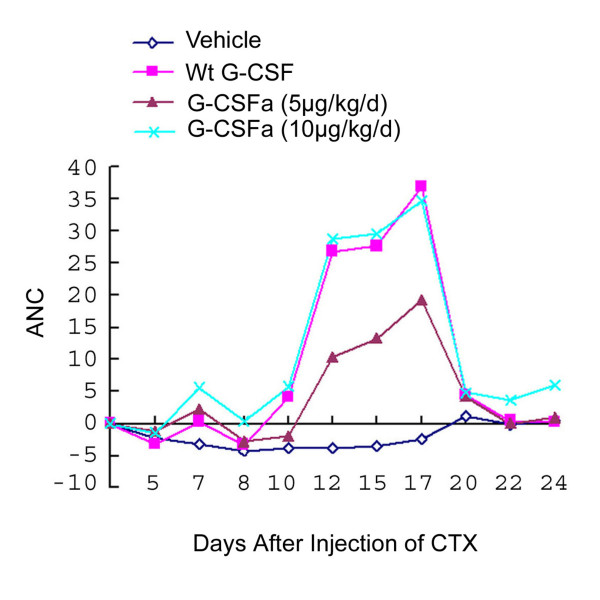
**Neutrophil recovery in neutropenia monkeys administered with G-CSFa and wild-type G-CSF**. Neutropenia monkeys were obtained by injection with CTX for 2 days as described in Materials and Methods. Five days after CTX injection, groups of monkey (n = 5) were administered daily via s.c. for 13 days. Peripheral blood absolute neutrophil counts (ANC) were determined at day 5, day 7, day 8, day 10, day 12, day 15, day 17, day 20, day 22, and day 24. The data were summarized from two independent experiments and similar results were obtained.

We next test the in vivo efficacy of G-CSFa in stimulating neutrophil production using the primate model. Anemic monkeys were obtained by the injection with cyclophosphamide (CTX) for 2 days at a dose of 50 mg/kg/day. Five days after CTX injection, G-CSFa was administered daily via s.c. for successive 13 days. Wild-type of G-CSF at the same dose was administered into separate groups of monkeys as a positive control. Absolute neutrophil counts (ANC) in peripheral blood were determined at various times post cytokine treatment. ANC in control monkeys treated with the vehicle remained low for almost three weeks before bouncing back to the pretreatment level (Figure 5). In contrast, both G-CSF and G-CSFa were able to reduce both the degree and the duration of neutropenia, which were characterized by a dual-peak curve of neutrophil increase. The first peak appeared at day 7 and the second peak between day 12 and day 17. At day 7, G-CSFa, but not G-CSF, induced a significant (37%) increase in neutrophils compared with the pretreatment level (Figure 5). Consistent with the mouse data, the effect of G-CSFa on neutrophil production lasted longer than that of G-CSF. After the cessation of cytokine administration at day 22, ANC in monkeys administered with G-CSFa (10 ug/kd/day), but not G-CSF (10 μg/kg/day), remained significantly above the pretreatment level with CTX.

**Figure 6:**
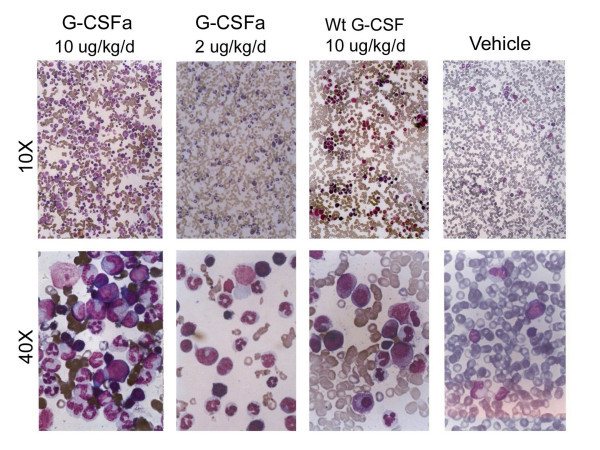
**Bone marrow cellularity of mice treated with G-CSFa or wild-type G-CSF**. Bone marrow cells from mice treated with G-CSFa or G-CSF were subjected to routine Wright staining and examined under a light microscope. Representative cell images at low magnification (10 ×) and high magnification (40 ×) are shown.

We next directly examined neutrophil production in bone marrow of monkeys undergone various treatments. Microscopic examination revealed that the level of nucleated cells in monkeys administrated with vehicle alone was very low, consistent with the neutropenic condition induced by CTX (Figure 6). However, treatment with G-CSF resulted in a significant increase in the number of nucleated cells, most of which belong to the neutrophil lineage. Consistent with the ANC kinetics shown above, G-CSFa also stimulated the production of nucleated cells in bone marrow and the stimulation was more potent than G-CSF at the same dosage. Morphological analysis indicated that these cells were primarily neutrophils of various differentiation stages.

## Discussion

G-CSF is a glycoprotein with a molecular mass of approximate 20 kDa. It is a bioactive molecule that has been extensively used in the clinic as a therapeutic agent for supporting the production of blood cells of the neutrophil linage [15]. It also displays biological effects on various aspects of hematopoiesis both in vivo and in vitro [8]. G-CSF has widely used in the clinic for over 15 years, primarily for accelerating neutrophil recovery in cancer patients with myelo-suppressive chemotherapy or radiotherapy [9,10]. G-CSF is a glycoprotein although glycosylation is not essential for its bioactivity. Clinical studies have demonstrated that recombinant non-glycosylated G-CSF expressed in and purified from *E. coli *displays almost the same therapeutic efficacy as glycosylated form of G-CSF [11,16]. Native G-CSF contains five cysteine residues. They form two internal disulfide bonds (Cys36-Cys42 and Cys64-Cys74), leaving one cysteine residue (Cys17) with a free sulfhydryl group. It is conceivable that this free cysteine residue may pose some problems during G-CSF purification and refolding. Firstly, the presence of Cys17 may increase the frequency of mismatch during the formation of intra-molecular disulfide bonds, resulting in a reduced yield of refolding. Secondly, it is possible that the free sulfhydryl group in cysterine residues may interfere with the stability of G-CSF. In other words, Cys17 may form inter-molecular disulfide bonds, resulting in the formation of G-CSF oligomers under certain oxidized condition. Oligomerization can conceivably lead to a decrease in the availability of G-CSF.

We reason that the substitution of cysteine 17 with alanine may result in an enhanced bioavailability and bioactivity of G-CSF, possibly through the elimination of oligomerization caused by the formation of inter-molecular disulfide bonds. In fact, our cell-based assays and in vivo studies in both mice and monkeys are consistent with the notion. Significantly, as evidenced from the examination of the first peak of neutrophil increase (Figure 5), G-CSFa induced a much higher level of ANC than G-CSF did. Although it is relative small this increase is of great value for patients receiving myelo-suppressive therapies. It is the period when patients are most susceptible to infections due to drastic neutrophil reduction. Therefore, a shortened window in which patients have low neutrophil counts will greatly facilitate them to combat deleterious infections. Further supporting this notion, a separate pharmacokinetic study reveals that G-CSFa exhibits both better stability in vitro and higher bioavailability in vivo than wild-type G-CSF [17].

G-CSF exerts its activity through the interaction with its receptor (G-CSF-R). Upon binding to G-CSF-R, G-CSF induces a signal transduction cascade in target cells, leading to various biological manifestations including cell proliferation and differentiation. G-CSF belongs to the long chain family of cytokines with an anti-parallel 4-helix bundles and long overhand loops. The major binding site on G-CSF has been shown to include residues in A and C helices [18,19]. Further studies indicates that Glu19 in the A helix of G-CSF molecule electrostatically inter-reacts with Arg288 of G-CSF-R [20,21]. A recent study on the crystal structure of G-CSF, complexed with the cytokine homologous region of G-CSF-R, reveals that residues in the amino-terminus of G-CSF may act as additional contact sites with G-CSF-R, which is unstructured in the unbound protein [22]. In this respect, the addition of arginine, glycine, and serine residues in the amino-terminus of G-CSFa that results in a more positive charge in the amino-terminus of G-CSFa may further enhance the binding between the cytokine and its receptor. This may also contribute to the higher bioactivity observed with the mutant G-CSF. It is conceivable that the tighter binding to its cognate receptor may render G-CSFa to be dissociated from its receptor at a slower rate, resulting in a longer time of action both in vivo and in vitro. In fact, G-CSF with a single amino acid substitution (Cys17 to Ala17) shows a better stability in plasma [17]. Therefore, we believe that the same mutation in G-CSFa also contributes to the enhanced bioactivities.

During the past decade or so, great efforts have been directed to finding a more stable and thus more effective G-CSF because of its instability in vivo. PEGylated G-CSF has been reported to enhance the stability of this cytokine. However, the steric hindrance effect of PEGylated proteins significantly suppresses the specific binding of PEGylated proteins to their cognate receptors or substrates [23]. Besides, pegylation calls for an additional modification step after obtaining purified target protein, which makes the production process inconvenient and adds costs to the production. In the current study, we report that G-CSFa exhibits an enhanced bioactivity due likely to its better stability. As a chronic toxicity study shows that G-CSFa does not exhibit significant toxicity and immunogenicity in rats [24], this cytokine derivative can be further explored for the development of a new generation of therapeutic agents for patients with neutropenia.

## Competing interests

The authors declare that they have no competing interests.

## Authors' contributions

YJ was involved in experimental designs, data acquisition and analysis data interpretation as well as drafting manuscript. YQ carried out protein purification experiments and was involved in data acquisition, analysis and interpretation. WJ conducted in vitro experiments including protein purification and analysis. WD was involved in the analysis and interpretation of data as well as manuscript preparation.

The authors read and approved the manuscript.
